# Pure intracorporeal laparoscopic radical cystectomy with orthotopic “U” shaped ileal neobladder

**DOI:** 10.1186/1471-2490-14-89

**Published:** 2014-11-18

**Authors:** Antonio Luigi Pastore, Giovanni Palleschi, Luigi Silvestri, Giuseppe Cavallaro, Mario Rizzello, Gianfranco Silecchia, Cosimo de Nunzio, Samer Fathi Al-Rawashdah, Vincenzo Petrozza, Antonio Carbone

**Affiliations:** Department of Medico-Surgical Sciences and Biotechnologies, Faculty of Pharmacy and Medicine, Urology Unit ICOT, Sapienza University of Rome, Via Franco Faggiana 1668, Latina, 04100 Italy; Department of Medico-Surgical Sciences and Biotechnologies, Faculty of Pharmacy and Medicine, Surgery Unit ICOT, Sapienza University of Rome, Latina, Italy; Department of Urology Unit, Sant’Andrea Hospital, Sapienza University of Rome, Rome, Italy; Department of Medico-Surgical Sciences and Biotechnologies, Faculty of Pharmacy and Medicine, Histopathology Unit ICOT, Sapienza University of Rome, Latina, Italy; Uroresearch Association, non-profit research, Latina, Italy

**Keywords:** Laparoscopic radical cystectomy, Orthotopic neobladder, Urinary diversion, Ileal neobladder, Bladder cancer

## Abstract

**Background:**

Radical cystectomy with pelvic lymph node dissection represents the standard treatment for muscle-invasive, and high-risk non-muscle-invasive bladder cancers. Aim of this study was to report our case series of 30 patients undergoing totally laparoscopic radical cystectomy (LRC) with reconstruction of an intracorporeal orthotopic ileal neobladder. Intra- and perioperative results and the functional and oncological outcomes 9 months after operation are reported.

**Methods:**

Between October 2010 and December 2012, 30 male patients underwent LRC with a pure laparoscopic orthotopic ileal “U”- shaped neobladder diversion. The men had a median age of 67 years, a median body mass index of 22.3, and a mean ASA score of 2.2; they represented various clinical stages of disease.

**Results:**

None of the patients required conversion to open surgery, and no perioperative mortalities were reported. The median operating time was 365 min, and the median blood loss was 290 mL, with a transfusion rate of 26.6%. All surgical margins were negative; 8 patients with non–organ-confined disease or positive lymph nodes received adjuvant chemotherapy. Early complications (within 30 days) occurred in 7 patients, and late complications occurred in 6 patients. The mean hospital stay was 9 days. At 9 months after surgery, the daytime continence rate was 83.3% and the nighttime continence rate was 73.3%.

**Conclusions:**

Pure LRC with intracorporeal orthotopic ileal neobladder reconstruction may represent a viable alternative to open radical cystectomy, with a significant reduction in patient morbidity. Future, large, randomized controlled trials with extensive follow-up are needed to confirm our encouraging results.

## Background

Radical cystectomy (RC) with pelvic lymph node dissection (PLND) and urinary diversion is still considered the gold standard treatment for muscle invasive bladder cancer [1]. To reduce morbidity and decrease the duration of hospitalisation, minimally invasive surgical approaches have been described since 1992, when laparoscopic radical cystectomy (LRC) was first reported [2, 3]. Technical difficulties and high costs have hampered the widespread adoption of this technique [4], but its broader use is being facilitated by the development of new dissection and haemostatic devices that allow precise topographic definition of the surgical field and which simplify some surgical steps. As a result, better oncologic and functional outcomes have been reported [5].

The purely intracorporeal reconstruction of an orthotopic neobladder was not performed until 2000 [6]. Since then, expertise with laparoscopic surgery has increased, with a greater interest in this procedure being developed and the consequent report of a variety of reconstructive options. LRC and robot-assisted RC (RARC) with different urinary diversion methods have been demonstrated to be feasible, safe, and capable of providing many operative and functional advantages [7, 8]. However, there are few reports describing the perioperative, oncologic, and functional outcomes in case series involving totally intracorporeal neobladder reconstruction. Herein, we report the results of LRC that involved a complete, intracorporeal, orthotopic, “U”-shaped, ileal neobladder in 30 patients, with a median follow-up period of 16.5 months (maximum, 32 months).

## Methods

Between December 2010 and December 2012, 30 consecutive patients underwent LRC with intracorporeal orthotopic neobladder reconstruction in our department. The study was performed according to the Ethical Principles for Medical Research Involving Human Subjects (World Medical Association, The Declaration of Helsinki Principles, 2000). A local ethical committee approval was obtained (ASL Lt/no.12834652/2010). Written informed consent was obtained from all patients. The inclusion criteria for this procedure included (1) muscle-invasive, urothelial bladder cancer T2–4a, N0–Nx, M0; (2) high-risk or recurrent non–muscle-invasive tumours or multifocal T1G3; and (3) T1G3 with concomitant carcinoma in situ (CIS). Patients were not eligible for this procedure according to the following criteria: (1) patient refusal of LRC with orthotopic diversion; (2) the presence of an LRC contraindication, including distant metastases, an American Society of Anaesthesiologists (ASA) score >3, severe heart and/or respiratory failure; and (3) the presence of contraindications to neobladder diversion, including the presence of an urethral tumour, urethral stricture, extensive abdominal surgery, or severely insufficient renal function. Relative contraindications were an age >75 years and a body mass index (BMI) >30.

### Surgical techniques

#### Cystoprostectomy

Each patient was placed in a supine, steep Trendelenburg position (20–25°), and pneumoperitoneum (12 mmHg) was established. A five-port, fan-shaped, transperitoneal approach was used, according to the Hasson technique [9]. Bilateral pelvic lymphadenectomy was performed as the first step. The boundaries of standard PLND were the bifurcation of the common iliac artery, proximally; the genitofemoral nerve, laterally; the circumflex iliac vein and lymph node of Cloquet, distally; and the hypogastric vessels, posteriorly, including the obturator fossa. Extended PLND (in order to reach the aortic bifurcation) was performed in all cT3 cases and when computed tomography revealed a pelvic lymphadenopathy.

**Figure 1:**
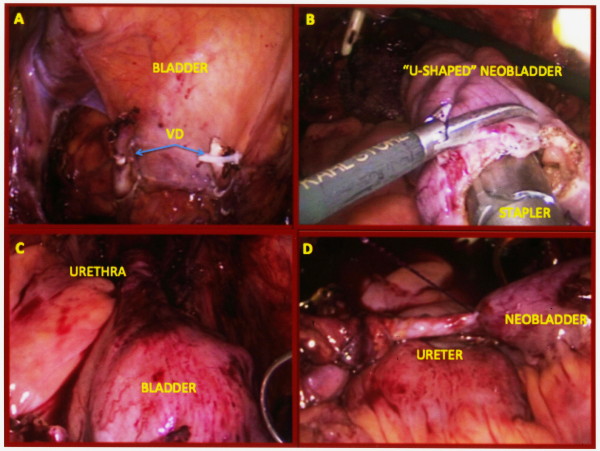
**Key surgical steps of cystectomy and ileal neobladder reconfiguration. A**. Seminal vesicles and vas deferens were dissected and maintained en bloc with the bladder, using a 5 mm Hem-o-lok. **B**. “U-Shaped” neobladder configuration with stapler device. **C**. The neoblader-urethral anastomosis is completed with two Filbloc running barbed 3–0 sutures. **D**. Neobladder-ureteral anastomosis with Filbloc running barbed 3–0 suture.

After PLND, a 7- to 8-cm transverse peritoneal incision of the pouch of Douglas was made with a 5-mm Ligasure (Covidien, Boulder, CO, USA). The umbilical ligaments and the urachus were divided, proximally, allowing entry into the space of Retzius to mobilise the bladder. The ampullae of the vas deferens were transected bilaterally, and the seminal vesicles were dissected and maintained, en bloc, with the bladder using a 5-mm Hem-o-lok (Teleflex Medical, Research Triangle Park, NC, USA) (Figure 1A). The Denonvilliers’ fascia was then incised, and a Denonvilliers’ space between the rectum and the prostate was developed. The endopelvic fascia was incised bilaterally, and the puboprostatic ligaments and the dorsal vein complex were dissected with the Ligasure device. The ureters were isolated, bilaterally, ligated, and transected with cold scissors, just outside the bladder; the distal margins of the ureters were also sent for frozen section biopsy. The lateral pedicles of the bladder and the prostate were bilaterally isolated and divided with Hem-o-lok. In cases where we attempted a nerve-sparing procedure, an intrafascial dissection was performed at the dorso-lateral part of the prostate. Hem-o-lok clips were used to avoid thermal injury to the neurovascular bundles during cautery. The apex of the prostate, the urethra and the recto-urethral muscles were divided using scissors.

#### Neobladder reconfiguration

A 40-cm segment of ileum, 20 cm from the ileocecal junction, was isolated, and ileo-ileal continuity was restored using Endo-GIA staplers (U.S. Surgical, Norwalk, CT, USA). The isolated ileal segment was detubularised and a globular U-shaped ileal neobladder was constructed (Figure 1B) and anastomosed to the urethra with two 15-cm, 3–0 barbed sutures (Filbloc 90 day absorbable suture, Assut Europe, Rome, Italy), using the Van Velthoven technique (Figure 1C) [10]. Each ureter was spatulated for a length of 2 cm and separately anastomosed to the terminal ends of the U-shaped ileal segment, using the Le Duc technique with continuous, 3–0 barbed sutures (Figure 1D) [11]. After suturing the posterior wall, two single-J, 7-Ch, 40-cm ureteral stents were inserted, using the Seldinger technique, through the abdominal wall, at the midline, just above the symphysis [12]. The stents were placed in the ureters through a 2-mm MiniPort trocar (Covidien), positioned just above the symphysis. The entire procedure was performed intracorporeally. The neobladder was irrigated to ensure a watertight closure; any leaks were secured with interrupted 2–0 Vicryl sutures. A suction drain was placed in the pelvis through a lateral port site.

The postoperative care included removal of the nasogastric tube on postoperative day 1; pouch irrigation every 8 hours starting on postoperative day 1; and removal of the abdominal drain when the output was <200 mL/day. The ureteral stents were removed 7 days postoperatively and a cystography control was obtained at 3 weeks and, if no leaks were observed, the catheter was removed.

## Results

### Demographic and operative data

The demographic data are summarized in Table 1. The 30 male patients had a median age of 67 years, a median BMI of 22.3, and a mean ASA score of 2.2. None of the patients had prior radiation treatment, 3 had received neoadjuvant chemotherapy, and 9 had failed intravesical instillation of bacillus Calmette-Guérin.

**Table 1:** **Patient demographics and clinical data**

Characteristic	Value
Age, median (range), years	67 (62–79)
BMI, median (range), kg/m^2^	22.3 (16.0–26.1)
ASA score, No. (%)	1) 9 (30)
	2) 15 (50)
	3) 6 (20)
Clinical stage, No. (%)	cT1, 12 (40)
	cT2, 16 (53.3)
	cT3, 2 (6.6)
Concomitant CIS, No. (%)	7 (23.3)
Primary tumour, No. (%)	16 (53.3)
Recurrence after operations with intravesical BCG therapy, No. (%)	9 (30)
Neoadjuvant chemotherapy No. (%)	3 (10)
Smoking history, No. (%)	21 (70)
Previous abdominal surgery, No. (%)	8 (26.6)
Other malignancies, No. (%)	3 (10)

BMI = body mass index; ASA = American Society of Anaesthesiologists;

BCG = Bacillus Calmette-Guérin; CIS = carcinoma in situ.

The operative data are presented in Table 2. None of the cases required conversion to open surgery, and no perioperative mortalities were reported. The median operating time was 365 min, with a median blood loss of 290 mL and a transfusion rate of 26.6%. The mean number of lymph nodes removed was 16 (range, 5–28). Overall, the median hospitalization duration and times to regular diet and ambulation were 9 (range, 7–37) days, 6 days, and 2 days, and, respectively.

**Table 2:** **Operative data**

Characteristic	Median value (range)
Operative time, min	365 (270–605)
Estimated blood loss, mL	290 (70–800)
Pelvic lymph nodes removed	16 (range, 5–28)
Transfusion rate, %	26.6
Time to liquid consumption, days	3 (2–7)
Time to regular diet, days	6 (4–11)
Time to ambulation, days	2 (2–4)
Length of hospital stay, days	9 (7–37)

### Pathologic data

The pathologic data are summarized in Table 3. All tumours were transitional cell carcinomas, but 30% (9/30) of the patients had concomitant carcinoma in situ; incidental prostate cancer was detected in 26.6% (8/30) of the patients. All surgical margins were negative, but 8 patients, with non–organ-confined disease or positive lymph nodes, received adjuvant chemotherapy.

**Table 3:** **Pathologic data**

Pathologic outcome	No. of cases (%)
**pT stage**	
***Organ confined disease***	
pT1	11 (36.6)
pT2	16 (53.3)
pT2a	11 (36.6)
pT2b	5 (16.6)
***Local extravesical disease***	
pT3	2 (6.6)
pT3a	1 (3.3)
pT3b	1 (3.3)
pT4a	1 (3.3)
**pN stage**	
pN-	25 (83.3)
pN+	5 (16.6)
**Grade**	
G1	4 (13.3)
G2	9 (30)
G3	17 (56.6)
**Concomitant CIS**	9 (30)
**Incidental prostate cancer**	8 (26.6%)
**Positive surgical margins**	0

### Complications

**Figure 2:**
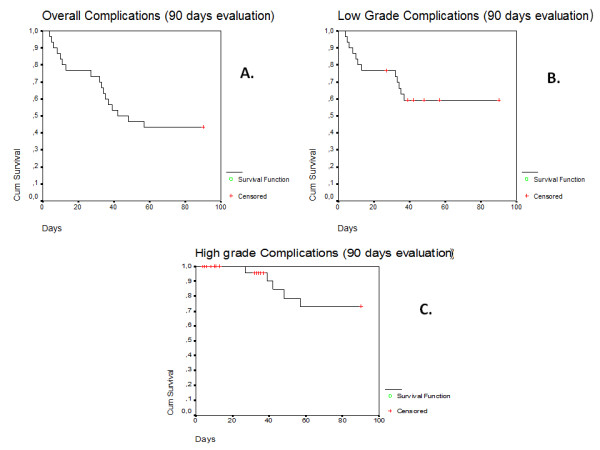
**90 day complications free survival in patients treated with laparoscopic radical cystectomy. A)** Overall; **B)** Low grade complications; **C)** High grade complications.

The complications that were encountered are presented in Table 4, along with their management. Complications were classified as early, if they occurred within the first 30 postoperative days, or late (>30 days after surgery). The complications were graded according to the modified Clavien classification system [13]. Early complications occurred in 8 patients (26.2%), including 7 presenting low-grade complications (Clavien grades 1–2) and 1 patient with high-grade complications (Clavien grades 3–5). Late complications occurred in 9 patients (Clavien grade 1, 3 patients; Clavien grade 2: 2 patients; Clavien grade 3a: 3 patients; Clavien grade 3b, 1: patient). Kaplan Meier curves were used to evaluate the occurrence of low and high grade complications. The overall and low/high grade complications free survival up to 90 days of follow-up were showed in Figure 2 (A, B, C). Low grade complication occurred more frequently during the first 20 days postoperatively (Figure 2B), and significantly earlier when compared to high grade complications (p = 0.001). All high grade complications occurred after at least 32 days (Figure 2C).

**Table 4:** **Complications, according to the modified Clavien classification system**

Grade	Complication	No. of cases (%)	Management
**Early (within 30 day of LRC)**		**8 (26.6)**	
**Low grade (1–2)**		**7 (23.3)**	
1	Wound infection	2	Antibiotics and bedside management
2	Delirium	2	Sedative
2	Deep venous thrombosis	1	Anticoagulation prolonged therapy
2	Bowel ileus	1	Conservative
2	Neobladder-urethral anastomosis leakage	1	Prolonged bladder catheterisation
**High grade (3–5)**		**1 (3.3)**	
3a	Uretero-pouch anastomosis stricture	1	Double-J indwelling stent
**Late (>30 d after LRC)**		**9 (30)**	
**Low grade (1–2)**		**5(16.6)**	
1	Chronic urinary retention	3	Intermittent self catheterisation
2	Deep venous thrombosis	1	Anticoagulation prolonged therapy
2	Pouchitis	1	Antibiotics
**High grade (3–5)**		**4 (13.3)**	
3a	Vesico-urethral anastomosis stricture	3	Endoscopic incision
3b	Ileal-pouch fistula	1	Reoperation

### Functional outcomes

At the 9-month post-surgical follow-up visit, the daytime continence rate was 83.3% (25/30 patients) and the nighttime continence rate was 73.3% (22/30 patients). The mean maximal neobladder capacity was 295 mL, with a mean post-void residual volume of 54 (range, 0–220) mL and a peak flow rate of 13.6 (9.7–33) mL/s. Three patients needed intermittent self-catheterization for chronic urinary retention (>150 mL).

### Follow-up data

The median follow-up duration was of 16.5 (range, 9–32) months, with 11 patients (36.6%) completing 2 years of follow-up. At the last outpatient visit (September 2013), 22 patients (73.3%) were alive without evidence of recurrence, 2 patients had both local recurrence and distant metastases, 1 patient had local recurrence, and 1 patient had distant metastasis. Five patients (16.6%) died from various causes: 3 from metastases (multiple sites) and 2 from causes unrelated to the bladder cancer (1 from myocardial infarction, 1 from a car accident).

## Discussion

Laparoscopic surgery has improved considerably over the last decade, with indications now including difficult and prolonged procedures, such as radical cystectomies. Since the first LRC was described in 2000, numerous centres have published their experiences with this procedure [6, 14]. However, very few centres have reported data for more than 50 LRCs. Therefore, open RC still represents the reference standard treatment for muscle invasive bladder cancer whilst LRC is quickly emerging as a viable and safe alternative in high volume centres. Minimally invasive robotic and laparoscopic approaches have shown technical promise and are being increasingly adopted, as comparisons of perioperative and pathologic features have demonstrated encouraging results [15, 16]. However, concerns about the long-term oncologic efficacy of RARC/LRC remain to be fully addressed with large, prospective, long-term, follow-up studies.

In the current study, we presented data regarding our experience with LRCs, involving pure intracorporeal neobladder diversions, all performed by one skilled laparoscopic surgeon assisted by the same surgical team. The collected results suggest that localized, muscle-invasive bladder cancer can be successfully managed by LRC. In fact, we achieved operating times, intra-operative blood losses, and hospitalisation times comparable with those reported in the largest published series; the overall complication rate was also similar to the data reported in the literature. The type of complications that were encountered confirmed the safety of the procedure. Specifically, 70% (12/17 patients) required conservative treatment, without further surgical intervention, and were classified as being “low-grade,” complications, based on their Clavien classification (1 and 2); the remaining 5 patients required re-operations (4 needed minimally invasive endoscopic procedures, and 1 required major open surgery due to an ileal pouch fistula that developed 56 days after LRC). The major complication rate reported in our study remains less than that for open RC procedures and was comparable to previously published experiences.

Haber et al. compared 25 consecutive LRCs with a contemporary cohort of 25 open RCs with urinary diversions (14 ileal conduits, 11 orthotopic neobladders). The operative times, blood losses, transfusion rates, and incidence of ileus were lower in the LRC group. Additionally, there were no significant differences between the groups with regard to oral intake, times to ambulation, postoperative complications, or hospitalization stays [17]. The largest LRC series, to date, reported by Huang et al., included 171 patients [18]. Their median operative time was 325 min, with a median blood loss of 270 mL. They reported a total complication rate of 39.2% (67/171), with 6.4% (11/171 patients) having Clavien I complications, 19.3% (33/171) having Clavien II complications, and 13.5% (23/171) having Clavien III complications. Considering that the other experiences reported in the literature describe more complications in patients who had orthotopic neobladder substitutions, as compared to ileal conduits, this would explain why Huang et al. reported a higher complication rate than that observed in other studies; they only performed neobladder procedures.

There is significant variability in the results reported regarding operative data, as well as complication rates. These data reflect the available surgical expertise and the volume of procedures conducted in the different centres, and highlights the absence of consensus regarding the operative procedures and urinary diversion techniques used in laparoscopic surgery. In a retrospective study, Aboumarzouk et al. compared 155 patients who underwent LRC or open RC, with a mean follow-up period of 53 months. In this trial, the open RC group had shorter operative times (p <0.0001), more blood loss, higher transfusion requirements (p <0.00001), and longer hospitalisations than did the LRC group. No differences were found regarding lymph node yields, positive margins, pathologic results, or positive lymph nodes. The open RC group experienced fewer intraoperative complications (p =0.03). Significant differences were not found between the two groups regarding 5-year overall survival, cancer-specific survival, and recurrence-free survival. The authors concluded that LRC may be considered an alternative to open RC with good operative results, in addition to comparable oncologic outcomes [19]. Another 2 recent systematic reviews evaluated 19 studies comparing open RC to LRC and RARC; they concluded that there is no evidence of superiority for one modality over the others [20, 21]. The authors also reported that despite the selected cohort of patients included in the LRC and RARC groups, compared to the non-selective ORC group, they were unable to show a significant impact on oncological outcomes. However, both reviews agreed that the LRC and RARC results are encouraging and supported the diffusion of these minimally invasive techniques.

New evidence seems to suggest that improved surgical skills and experience helps with the replacement of open RC with LRC. Robotic techniques are rapidly replacing laparoscopy due to the shorter learning curve and similar, if not better, operative and post-operative results. In a prospective, comparative analysis between LRC and RARC procedures involving ileal conduit urinary diversions, Abraham et al. reported that both LRC and RARC cystectomies can be safely performed without compromising oncologic standards for surgical margins or the extent of lymphadenectomy. The robotic approach appears to have a shorter learning curve, and is associated with less blood loss, fewer complications, and an earlier return of bowel function than is LRC [22]. Furthermore, 2 prospective, controlled randomised trials, comparing RARC to open RC, noted that RARC was associated with a significantly decreased estimated blood loss, and a reduced transfusion rate, operative time, and analgesic use. No significant differences were observed regarding complications, length of hospital stay, or pathological outcomes [23, 24].

In our experience, pure LRC combined with orthotopic neobladder creation in male patients with invasive bladder carcinoma results is safe and feasible in terms of radical tumour resection, and offers a minimally invasive procedure with a quick recovery. With the improvement of laparoscopic techniques and the development of innovative equipment [5], the treatment of bladder cancer using pure LRC and the creation of orthotopic neobladders is maturing. Compared to the open procedure, this technique has 3 principal advantages. First, the technique offers a clearer operative view, which facilitates meticulous dissection and reduces injury to the pelvic floor structure, thus improving the early postoperative recovery of continence. Second, each dissection is performed with good hemostasis, and the pneumo-peritoneal pressure can also decrease venous haemorrhage. Third, the bowel is never exposed extracorporeally. We consider that the reduced invasiveness of the bowel manipulation was associated, in our case series, with quicker functional recovery of the intestinal tract and decreased the incidence of related postoperative complications. This experience also addresses the concept that LRC, with totally intracorporeal urinary diversion, is a procedure for skilled surgeons and that significant laparoscopic experience is important to reduce the operative time. Furthermore, the surgical strategy and the selection of specific devices are crucial to simplify some of the critical steps of the procedure, such as ureteral stenting and uretero-neobladder anastomosis. These aspects are essential to shortening the operation, which is mostly dedicated to the reconstructive phase. Based on our experience, good patient selection and a highly standardized procedure, managed by a skilled surgical team, allows the achievement of acceptable operative times and good oncologic and functional outcomes. This experience may be reproduced in many other institutions where expert surgeons have an extensive laparoscopic background. Experienced surgeons, assisted by expert teams, may reduce the operative time for the configuration of the intracorporeal neobladder to balance the time necessary to restore body integrity by suturing the peritoneum, fascia, muscles, and skin during an open procedure. These results should be supported from experiences involving large cohorts of patients.

The main limitation of this study is the relatively small sample size and the follow-up duration. However, the primary aim of this study was to report our experience with the technique, performed by one surgical team. Nevertheless, a larger, multi-institutional analysis with a longer follow-up period is needed to confirm that this LRC approach, with open neobladder configuration, achieves long-term oncologic and functional outcomes superior to those achieved using open RC.

## Conclusions

Pure LRC, with intracorporeal orthotopic ileal neobladder reconstruction, may represent a viable alternative to open radical cystectomy with a significant reduction in patient morbidity. Future large, randomized controlled trials with extensive follow-up are needed to confirm and update the results of this study.

## Abbreviations

ASA: American Society of Anesthesiologists
BMI: body mass index
CIS: Carcinoma in situ
LRC: Laparoscopic radical cystectomy
PLND: Pelvic lymph node dissection
RARC: Robot-assisted radical cystectomy
RC: Radical cystectomy.
